# Pooled Analysis of Trabectedin Efficacy in Myxoid/Round Cell Liposarcoma from Three Prospective Clinical Trials

**DOI:** 10.3390/cancers18121921

**Published:** 2026-06-12

**Authors:** Michael Jason Nathenson, Lucy Hoch, Beth Ireland, Rose O’Nians Michalowicz, Dennis Williams, Neeta Somaiah

**Affiliations:** 1 US World Meds, Louisville, KY 40241, USA; rose.onians@adaptimmune.com; 2 Adaptimmune, Oxfordshire OX14 4SB, UK; lucy.hoch@gmail.com (L.H.); bethkireland@gmail.com (B.I.); 3 Verismo Therapeutics, Philadelphia, PA 19104, USA; dennis.williams@verismotherapeutics.com; 4 Department of Sarcoma Medical Oncology, UT MD Anderson Cancer Center, Houston, TX 77030, USA; nsomaiah@mdanderson.org

**Keywords:** sarcoma, soft tissue sarcoma, trabectedin, myxoid liposarcoma, myxoid/round cell liposarcoma, pooled analysis

## Abstract

Background/Objectives: Trabectedin is a standard-of-care chemotherapy for recurrent/metastatic liposarcomas, including myxoid/round cell liposarcoma (MRCLS). This pooled analysis was performed to evaluate trabectedin efficacy in MRCLS across multiple trials to establish a standard-of-care efficacy baseline. Methods: The Yale University Open Data Access (YODA) Project was queried for prospective trabectedin trials. Data on patients with unresectable, locally advanced/metastatic MRCLS who received ≥ 1 trabectedin dose(s) (1.0–1.5 mg/m^2^ administered as a 24-h continuous infusion every 3 weeks) were extracted from three trials: NCT00060944, NCT00210665, and NCT01343277. The primary outcome was the objective response rate as per the Response Evaluation Criteria in Solid Tumors (RECIST) v1.1. Secondary outcomes included disease control rate and overall survival, defined as the time from first trabectedin administration to death. Responses were assessed using RECIST by investigators in NCT01343277 and by independent review in NCT00060944. In NCT00210665, tumor assessment followed institutional standards, so only overall survival was analyzed. Results: Sixty-three patients were included (42 from NCT01343277, 13 from NCT00060944, 8 from NCT00210665): 32% were female, the median age was 50 years, and 79% were White. The objective response rate was 16.3% (8/49) of patients (37 from NCT01343277, 12 from NCT00060944). The disease control rate was 77.6% (38/49). Overall survival data were available for 63 patients: the median overall survival was 22.51 months (95% CI: 16.99–34.33). Conclusions: This analysis represents one of the largest pooled populations to date of patients with advanced MRCLS treated with trabectedin in prospective clinical trials to be reported. Whilst limited by typical factors affecting pooled analyses, including cross-study heterogeneity, nevertheless the results estimate a trabectedin efficacy baseline against which to inform other therapies for MRCLS.

## Simple Summary

Trabectedin is an effective, standard-of-care chemotherapy for recurrent or metastatic tumors after initial treatment for the soft tissue cancer, myxoid/round cell liposarcoma (MRCLS). It can replace more toxic agents used earlier to shrink tumors and improve response and survival in patients who have already received previous chemotherapy. Current benchmarks for trabectedin effectiveness are limited. Thus, an MRCLS standard-of-care baseline for trabectedin effectiveness was created based on three MRCLS clinical trials available in the Yale University Open Data Access Project database. Sixty-three patients with advanced MRCLS that could not be removed surgically survived for 22.5 months, 16.3% responded to trabectedin treatment, and 77.6% experienced disease control. As one of the largest MRCLS groups studied, this data enables better estimates of trabectedin’s benefits and a standard-of-care baseline against which to compare novel or future therapies.

## 1. Introduction

Trabectedin was approved by the US Food and Drug Administration in 2015 as a systemic chemotherapy treatment for recurrent or metastatic liposarcoma and leiomyosarcoma [1,2]. Trabectedin is considered to be standard-of-care for patients with recurrent or metastatic liposarcoma or leiomyosarcoma that has progressed after standard anthracycline-based chemotherapy. Liposarcoma includes genetically distinct subtypes: well-differentiated/de-differentiated, pleomorphic, and myxoid (with varying proportions of round cell) liposarcoma (MRCLS). Approximately 30% of liposarcomas are MRCLS [3]. The age-adjusted incidence rates of MRCLS are approximately 0.21 and 0.12 cases per 100,000 p-years in the United States and Europe, respectively [4,5], equating to approximately 1400 new cases of MRCLS per year across these regions.

Typical prognostic factors for MRCLS include size, grade, and tumor location. Low-grade tumors have a lower chance of recurrence; however, intermediate- or high-grade tumors have an approximately 50% chance of recurrence despite local therapy comprising surgery and radiation [6]. The median overall survival for metastatic MRCLS is only approximately 10 to 30 months [7,8,9], resulting in a population of metastatic MRCLS with limited treatment options and a high unmet need for additional therapies.

The malignancy is characterized by chromosomal translocations resulting in fusion of the deoxyribose nucleic acid (DNA) Damage-Inducible Transcript 3 (*DDIT3*) gene (encoding the C/EBP Homologous Protein, CHOP) with the Fused in Sarcoma (*FUS*) or the Ewing Sarcoma Breakpoint Region 1 (*EWSR1*) genes, and is biologically distinct from other liposarcoma subtypes [10]. By contrast, well-differentiated and de-differentiated liposarcomas are characterized by amplification of chromosome 12q leading to amplification of Mouse Double Minute 2 (MDM2) and Cyclin-Dependent Kinase 4 (CDK4) amplification, and pleomorphic liposarcomas are genetically complex [10]. Given their rarity, clinical trials often group liposarcoma subtypes together and do not typically report comprehensive outcomes for individual, specific liposarcoma subgroups.

Trabectedin is a marine-derived tetrahydroisoquinoline alkaloid that exerts its antitumor activity through a unique mechanism of binding to the minor groove of DNA, inducing DNA damage, cell-cycle arrest, and, consequently, interference with transcriptional regulation [11]. Preclinical studies have demonstrated that trabectedin selectively displaces *FUS-DDIT3* from its DNA-binding sites, leading to sustained inhibition of its transcription-factor function and restoration of adipocytic differentiation pathways [12,13]. In parallel, trabectedin modulates the tumor microenvironment by selectively inducing apoptosis in monocytes and tumor-associated macrophages, thereby attenuating pro-tumorigenic inflammatory signaling and potentially enhancing anti-tumor immune responses [14]. Collectively, these mechanisms provide a compelling biological rationale for the exceptional and durable clinical activity of trabectedin in MRCLS.

In the phase III trial (NCT01343277) that compared trabectedin with dacarbazine in patients with previously treated advanced liposarcoma or leiomyosarcoma, the objective response rates were 9.9% with trabectedin and 6.9% with dacarbazine, and there were no complete responses in either group [1]. The median overall survival did not differ significantly between trabectedin (12.4 months) and dacarbazine (12.9 months) (Hazard Ratio, HR = 0.87; *p* = 0.37) [1]. The primary study endpoint of median progression-free survival in the overall cohort was significantly different with trabectedin and dacarbazine (4.2 and 1.5 months, respectively; HR = 0.55, *p* < 0.001) [1]. The greatest benefit, however, was observed in the subgroup of 57 patients with MRCLS, where the progression-free survival was 5.6 months with trabectedin versus 1.5 months with dacarbazine (HR = 0.41) [1]. The objective response rate and overall survival in the MRCLS subgroup were not, however, reported [1].

Trabectedin has also been studied in other subtypes of sarcoma, particularly translocation-associated sarcomas, including synovial sarcoma [15,16]. In a pooled analysis of two phase II trials of trabectedin in patients with advanced translocation-related sarcomas, the median progression-free survival was longer (7.4 months; 95% confidence interval, CI: 5.6–11.1 versus 5.6 months; 95% CI: 4.1–7.3) and the response rate was highest (27% versus 12%) in the subgroup of 22 patients with MRCLS than in the overall translocation-associated sarcoma population [17]. This suggests that MRCLS has a distinct sensitivity to trabectedin compared to other sarcomas, including leiomyosarcoma and other liposarcoma subtypes. Retrospective and real-world studies further support the efficacy of trabectedin in MRCLS, with reported response rates ranging from 15% to 51% [16,18,19,20,21,22,23]. However, these studies are limited by the biases inherent in retrospective and real-world data, including observation bias, sample size, single institution, and lack of standardized response criteria.

Additionally, other studies have also examined the utility of trabectedin in earlier lines of therapy for MRCLS, specifically neoadjuvant chemotherapy for localized disease, and they suggest that trabectedin may have similar efficacy to neoadjuvant anthracycline and ifosfamide with an improved safety profile [24,25,26]. A phase II trial of neoadjuvant trabectedin in 23 patients with advanced localized MRCLS reported an objective response rate of 24% (95% CI: 10–44) with no disease progression and a pathologic complete response rate of 13% (95% CI: 3–34) [24]. An expanded cohort from the neoadjuvant histology-tailored chemotherapy trial showed an overall survival of 88% at 60 months for trabectedin compared to 90% for standard (epirubicin/ifosfamide) chemotherapy (HR 1.2; 95% CI: 0.37–3.93; log-rank *p* = 0.77) [27]. Of the 36 patients who received trabectedin and were evaluable for response in this study, the overall response rate was 11.1%, with all experiencing partial responses and 88.9% having stable disease [27]. These studies highlight the potential value of trabectedin in the neoadjuvant setting for patients at a high risk of disease recurrence and offer a less toxic alternative to anthracycline/ifosfamide chemotherapy.

Overall, these studies highlight the effectiveness of trabectedin for MRCLS. A progression-free survival of 5.6 months from the phase III trabectedin versus dacarbazine trial can be used as a benchmark for comparison of future trials using this as the primary endpoint [1]. However, if the objective response rate or overall survival are used as trial endpoints, such benchmarks currently are derived only from retrospective data. The objective response rate is a valuable endpoint to consider in phase I or phase II soft tissue sarcoma trials, especially when a randomized trial with a survival endpoint may not be viable due to the low target population incidence [4,5]. Moreover, overall survival is an important endpoint to consider in a soft tissue sarcoma trial because a progression-free survival benefit does not always correlate with an overall survival benefit [28]. The aim of this pooled analysis, therefore, was to evaluate the efficacy of trabectedin in MRCLS across multiple trials to serve as an objective response and overall survival standard-of-care efficacy baseline for MRCLS against which future therapies could be compared.

## 2. Materials and Methods

### 2.1. Data Sources, Search Criteria, and Patient Population

A comprehensive literature search was performed to identify prospective clinical trials evaluating trabectedin in MRCLS using PubMed and Europe PMC, and “trabectedin”, “myxoid”, and “MRCLS” as keywords. The only prospective clinical trials evaluating trabectedin in soft tissue sarcomas identified were those found in the Yale University Open Data Access (YODA) Project, except for the histology-tailored neoadjuvant chemotherapy clinical trial in high-grade soft tissue sarcoma [27]. A research proposal to access patient-level data from these prospective clinical trials was submitted to and accepted by the YODA Project. Subsequently, a search of the YODA Project for prospective clinical trials evaluating trabectedin was conducted.

**Figure 1:**
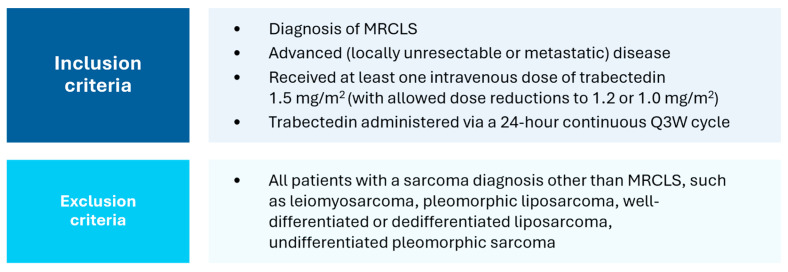
Criteria defining patient inclusion and exclusion in the pooled analysis. MRCLS, myxoid/round cell liposarcoma; Q3W, every 3 weeks.

The criteria used to define the patient population included in this analysis are shown in Figure 1, including the inclusion and exclusion criteria, as well as the method of trabectedin administration. Eligible patients had a confirmed diagnosis of myxoid liposarcoma MRCLS, unresectable (locally advanced) or metastatic disease. They had received at least one dose of intravenous trabectedin at the standard dose of 1.5 mg/m^2^. Subsequent dose reduction to 1.2 or 1.0 mg/m^2^ was allowed. Study data were mined to identify patients with a diagnosis of MRCLS, the relevant, pre-defined population for analysis. The terms “round cell”, “myxoid”, and “MRCLS” were used to identify patients in scope for the integrated analysis. Patients with a sarcoma diagnosis other than MRCLS, such as leiomyosarcoma, pleomorphic liposarcoma, well-differentiated or de-differentiated liposarcoma, and undifferentiated pleomorphic sarcoma, were excluded. Only patients who underwent the standard trabectedin administration method (a 24-h intravenous continuous infusion every 3 weeks) were included.

**Figure 2:**
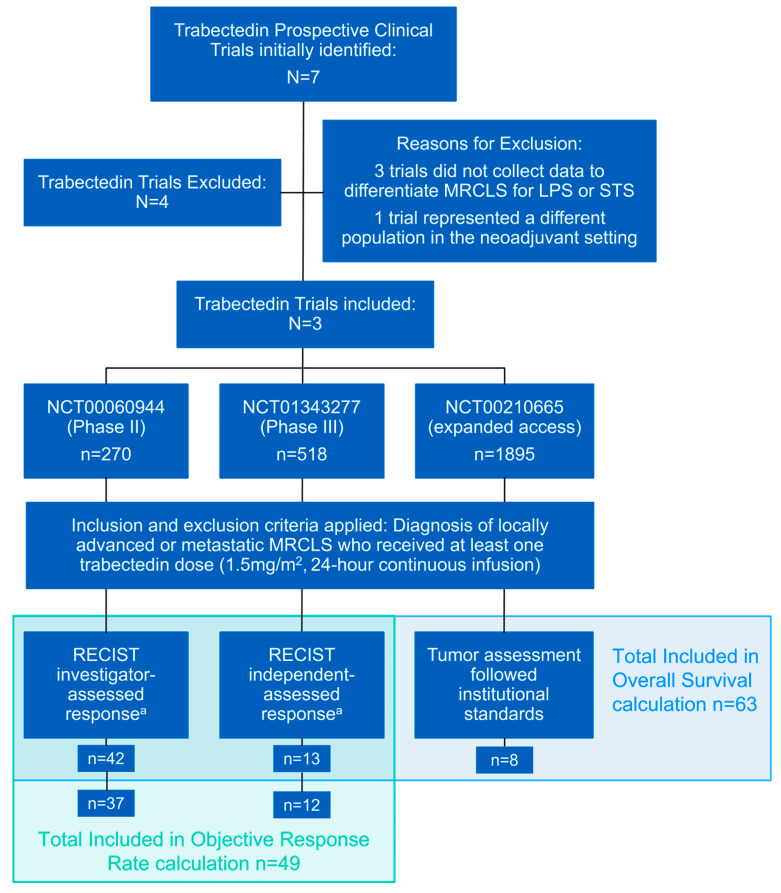
Flow diagram of trabectedin prospective clinical trials included in the pooled analyses. ^a^ Investigator- or independent-assessed tumor response by radiographic imaging of the chest, abdomen, and pelvis every 6 weeks for the first 36 weeks on study, and every 9 weeks thereafter until disease progression, subsequent anticancer therapy, or patient death occurred. LPS, liposarcoma; MRCLS, myxoid/round cell liposarcoma; N, number of studies; n number of patients; ORR, objective response rate; OS, overall survival; RECIST, Response Evaluation Criteria in Solid Tumors; STS, soft tissue sarcoma.

Seven prospective trabectedin clinical trials were identified, although four were excluded: one neoadjuvant trial that represented a different study population (localized resectable MRCLS) and three with no data to enable differentiation between MRCLS and other liposarcoma or sarcoma subtypes. The three trials included were a randomized, open-label, phase II trial comparing two different trabectedin dosing schedules (NCT00060944) [29]; a randomized, open-label, phase III trial of trabectedin versus dacarbazine in liposarcoma and leiomyosarcoma (NCT01343277) [1]; and an expanded-access protocol for trabectedin in leiomyosarcoma and liposarcoma (NCT00210665) (Figure 2). None of these trials were blinded. A retrospective, pooled analysis of individual participant-level data from locally unresectable or metastatic patients with MRCLS who had received at least one trabectedin dose and were enrolled in these three trials was performed. Since this was a secondary analysis of previously reported clinical trials, no sample size or power calculations were performed; the analysis was exploratory, leveraging the available data in this rare population to maximize statistical power.

### 2.2. Study Outcomes and Analysis

Demographic characteristics were analyzed using descriptive statistics. The primary outcome was the objective response rate, defined as the proportion of patients achieving a confirmed complete or partial response per the Response Evaluation Criteria in Solid Tumors (RECIST) v1.1 [30]. Secondary outcomes included disease control rate, defined as the proportion of patients achieving complete response, partial response, or stable disease; duration of response, defined as the time from the first documented response (complete or partial) until documented disease progression or death (whichever occurred first) as per RECIST v1.1; duration of stable disease, defined as the interval from stable disease to the first observation of disease progression in patients whose best response was confirmed stable disease as per RECIST v1.1 (among patients with available data); and, overall survival, defined as the time from first date of trabectedin administration to death or the censoring date. All three trials were included in the overall survival calculation, as shown in Figure 2; however, only prospective RECIST v1.1 response data were available from the phase II (NCT00060944) and phase III (NCT01343277) trials.

Response assessments were conducted every 6 weeks as per the respective trial protocols. Tumor responses were evaluated based on RECIST v1.1, as specified in the respective trial protocols. Objective response rate and disease control rate were estimated using the two-sided Clopper–Pearson exact binomial method to calculate the associated 95% CIs, which is appropriate for a small sample size [31]. Time-to-event endpoints (overall survival and duration of response) were evaluated using the Kaplan–Meier approach, allowing estimation of survival probabilities over time, with corresponding 95% CIs derived using the Brookmeyer–Crowley method [32,33]. Duration of stable disease was analyzed using descriptive statistics. Coding for the analyses and presentation was carried out using R (the ‘tm’ package), utilizing a double programming approach for quality control and reproducibility, in line with industry standards. A text mining approach was used to identify relevant data points.

## 3. Results

### 3.1. Baseline Demographics and Patient Characteristics

Patient numbers and baseline characteristics are shown in Table 1. Eligibility criteria for the three source trials required that all patients were intolerant to, or had relapsed or progressed after, previous standard-of-care cytotoxic chemotherapy (typically anthracycline and/or ifosfamide), including its administration in the neoadjuvant/adjuvant setting. Forty-nine patients were available for the objective response rate analysis: 37 (75.5%) from NCT01343277 and 12 (24.5%) from NCT00060944. Sixty-three patients were available for the overall survival analysis: 42 (66.7%) from NCT01343277, 13 (20.6%) from NCT00060944, and 8 (12.7%) from NCT00210665. The median age was 50 years (ranging from 25 to 75 years) for the objective response rate population, and a median age of 50 years (ranging from 20 to 75 years) for the overall survival population. The race distribution was also similar for the objective response rate and overall survival populations, with White (Caucasian) being the most common (77.6% and 79.4%, respectively). Additionally, the gender distribution was similar, with females comprising 34.7% of the objective response rate population and 31.7% of the overall survival population.

### 3.2. Objective Response Rate Analysis

The objective response rate was analyzed in 49 patients (Table 2). The objective response rate was 16.3% (8/49) with a 95% CI of 7.3 to 29.7. There were no complete responses; all eight responses were partial. The disease control rate was 77.6% (38/49) with a 95% CI of 63.4 to 88.2, and with 30 patients (61.2%) achieving stable disease. Among the seven patients with data available for duration of response, the median value was 7.66 months (95% CI: 0.03–10.35). Among the 30 patients with stable disease, only 22 had available data for duration of stable disease; of these, 18 (82%) had stable disease lasting 12 weeks or longer, and four (18%) had stable disease lasting less than 12 weeks. The median duration of stable disease among patients with stable disease lasting ≥12 weeks was 6.41 months (range: 0.03–15.740).

### 3.3. Overall Survival Analysis

**Figure 3:**
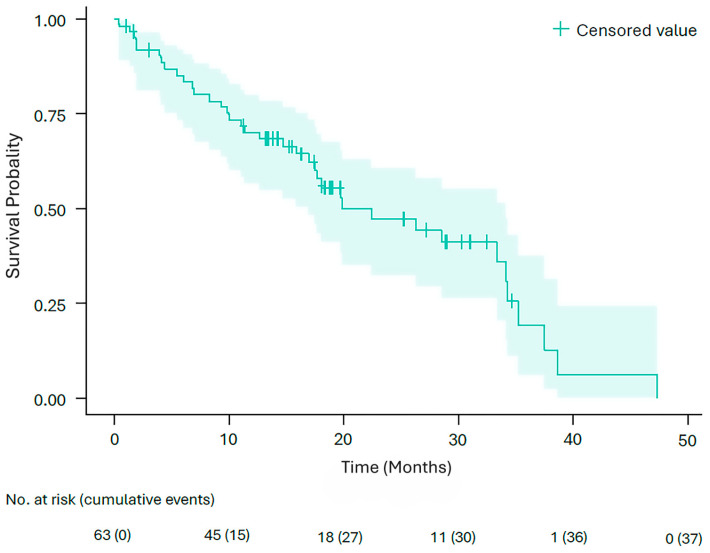
Kaplan–Meier curve showing the overall survival of the 63 patients with unresectable or metastatic myxoid/round cell liposarcoma receiving trabectedin in three prospective clinical trials (NCT00060944 [28], NCT00210665, and NCT01343277 [1]). The shaded area denotes the 95% confidence interval.

Kaplan–Meier analysis of overall survival was performed for 63 patients from all three prospective trabectedin clinical trials (Figure 2). The median overall survival from the time of the first trabectedin dose was 22.51 months (95% CI: 16.99–34.33). The Kaplan–Meier overall survival curve is shown in Figure 3.

## 4. Discussion

Trabectedin is approved for patients with unresectable or metastatic leiomyosarcoma or liposarcoma, including all liposarcoma subtypes, who have previously received anthracycline-containing regimens [1,2]. The liposarcoma subtypes include well-differentiated and de-differentiated liposarcoma, pleomorphic liposarcoma, and MRCLS. Trabectedin is considered particularly effective in MRCLS, with a median progression-free survival of 5.6 months [1] and real-world and retrospective series reporting response rates of 15% to 51% [17,18,19,22,23,24]. This efficacy is likely related to the trabectedin displacement of the DDIT3 fusion transcription factor from its DNA binding sites [13]. Occasionally, patients may achieve responses lasting several years [21]. Real-world response rates can be biased as response is not always assessed according to standard RECIST v1.1 criteria, with partial response defined as >30% reduction in the sum of lesion diameters. Instead, real-world response assessments may include any degree of tumor shrinkage. Thus, real-world data are a poor benchmark upon which to base comparisons of future therapies or clinical trials.

The utility of trabectedin in the neoadjuvant setting for high-risk localized disease has also been investigated, with objective response rates of 11.1–24%, pathologic complete response rates of 13%, and stable disease in 88.9% of patients being reported [24,26,27]. The clinical trial that examined different neoadjuvant histology-tailored approaches suggests that trabectedin has similar efficacy and reduced toxicity compared to anthracycline/ifosfamide for MRCLS in the neoadjuvant setting [27]. However, the absence of a control arm of radiation and surgery alone in this trial limits the assessment of the impact of neoadjuvant trabectedin on overall survival.

This comprehensive literature search identified only seven prospective clinical trials of trabectedin in soft tissue sarcomas. Patient-level data were available in the YODA Project, and a research proposal for this pooled analysis of trabectedin efficacy in MRCLS from prospective clinical trials was submitted to and subsequently approved by the YODA Project. To date, this represents one of the largest prospective datasets of patients with advanced MRCLS treated with trabectedin in prospective clinical trials, complementing previous, larger retrospective series of trials [19,22]. The patients were treated in two prospective clinical trials with trabectedin (NCT00060944 and NCT01343277) [1,29] for RECIST response assessment and in the prospective expanded-access protocol (NCT00210665) for additional overall survival assessment. They received at least one dose of trabectedin, i.e., 1.5 mg/m^2^ administered as a continuous intravenous infusion over 24 h, which is the current standard-of-care dosing, and were assessed for response as per RECIST v1.1 [30]. The objective response rate was 16.3%, with a median duration of response of 7.66 months, while the disease control rate was 77.6%, and 82% of patients had stable disease lasting longer than 12 weeks. Although the percentage of MRCLS patients with partial response to trabectedin was notably smaller in this analysis than in the real-world, retrospective series [19], the disease control rate was comparable, suggesting that the disparity in the reported response rates may be related to how a response assessment is defined, i.e., as per RECIST as opposed to clinical response.

This analysis further confirms the higher effectiveness of trabectedin for MRCLS specifically, with an objective response rate of 16.3% compared to the objective response rate of 9.9% reported in the randomized phase III trial of trabectedin versus dacarbazine in the total liposarcoma/leiomyosarcoma population [1]. Additionally, it provides an assessment of the objective response rate specifically in MRCLS based on prospective clinical trial data with a RECIST v1.1 assessment that can potentially serve as a benchmark for comparison with future therapies or clinical trials. The median overall survival in this analysis of 22.5 months compares favorably to that reported in the phase III trial for the entire liposarcoma/leiomyosarcoma population (12.4 months) [1] and in the phase III trial of eribulin versus dacarbazine in leiomyosarcoma/liposarcoma (13.5 months) [34]. These trials [1,34] were conducted in a population that had received prior anthracycline-based chemotherapy. The overall survival for all soft tissue sarcomas in the first-line metastatic setting is only ~21 months [35], raising the possibility that MRCLS may have better survival overall compared to other sarcoma subtypes. A multicenter, retrospective series suggests this same trend, with a median overall survival for MRCLS in the first-line setting of 29.9 months and 27.5 months the in second-line setting [7], which is similar to the 22.5 months reported here.

This pooled analysis is limited by factors intrinsic to such analyses, including a risk of selection bias, pooled methodology, missing data in the source trials, heterogeneity across studies (each of which had a different design, including different response assessments), treatment durations, and patient populations, as well as sample-size limitations. Additionally, this analysis is limited given the sparsity of prospective trabectedin clinical trials and the heterogenous nature of those that do exist. One trial that examined trabectedin in the neoadjuvant setting was excluded because this represented localized, resectable disease rather than unresectable or metastatic MRCLS, i.e., a different treatment population. Moreover, there were differences in data collection among trials, and three were excluded because data that would enable MRCLS to be differentiated from other liposarcoma or soft tissue sarcoma subtypes were not collected. Consequently, there was bias due to potential MRCLS patients being excluded from this analysis because they could not be appropriately identified retrospectively.

A further limitation was the lack of collection of a central response assessment in the trabectedin expanded-access protocol. Thus, MRCLS patients could only be included in the overall survival analysis and not the objective response rate analysis. Additionally, the RECIST response assessment collection differed between independent and investigator reviews, depending on the specific trial. These limitations resulted in a sample size for RECIST response assessments of only 49 patients; however, all 49 patients were identified as having MRCLS, had received at least one dose of trabectedin at the standard-of-care dose (1.5 mg/m^2^) and by the standard method of administration (24-h continuous infusion), and had patient-level data for prospective RECIST v1.1 response assessments, representing, therefore, a homogenous population for analysis.

Despite its limited size given the rarity of soft tissue sarcomas and MRCLS in particular, this analysis is among the largest, pooled MRCLS populations to be reported. The larger sample size increases statistical power and reduces the 95% CIs for these results compared to previous reports with even smaller sample sizes [18,22]. The variation in clinical trial treatment protocols was limited by analyzing only those patients who received the current trabectedin standard-of-care dosing (1.5 mg/m^2^, 24-h continuous infusion). Pooling across trials improves the ability to generalize the results to the general population. Despite the aforementioned clear limitations, the detailed efficacy outcomes presented here provide supportive evidence for the benefit of trabectedin in MRCLS, as well as estimates of objective response rate and overall survival that can inform comparisons with future therapies or clinical trials.

## 5. Conclusions

The number of systemic chemotherapy options available to patients with soft tissue sarcomas, and especially MRCLS, is limited. Myxoid/round cell liposarcoma is particularly responsive to trabectedin chemotherapy, and trabectedin is considered to be a standard-of-care treatment in patients with unresectable or metastatic MRCLS previously treated with anthracycline-based chemotherapy. In fact, trabectedin is currently the preferred option for MRCLS in the second-line metastatic setting. Given the efficacy of trabectedin in MRCLS, future comparative clinical trials should use trabectedin in the control arm. Unfortunately, a benchmark for the efficacy (objective response rate or overall survival) of trabectedin specifically in MRCLS is currently inadequate, as these benchmarks are based on real-world or retrospective data. This pooled analysis represents one of the largest populations to date of patients with advanced MRCLS treated with trabectedin in prospective clinical trials. Despite the small sample size, the strength of the inclusion and exclusion criteria utilized in this analysis did result in a homogenous and generalizable population for analysis. Whilst limited by factors intrinsic to pooled analyses, such as heterogeneity across studies, selection bias, the small sample size, and heterogeneity of RECIST assessments, the results nevertheless estimate a trabectedin efficacy baseline (objective response rate and overall survival) against which to compare other or future therapies for MRCLS.

## Figures and Tables

**Table 1:** Baseline Demographics and Characteristics.

	Analysis	
	Objective Response Rate n = 49	Overall Survival N = 63
Patients from each trial, n (%)		
NCT01343277 [1]	37 (75.5)	42 (66.7)
NCT00060944 [29]	12 (24.5)	13 (20.6)
NCT00210665	-	8 (12.7)
Age, median (range), years	50 (25–75)	50 (20–75)
Female, n (%)	17 (34.7)	20 (31.7)
Race, n (%)		
White	38 (77.6)	50 (79.4)
Black or African American	7 (14.3)	8 (12.7)
Asian	2 (4.1)	3 (4.8)
Other/unknown	2 (4.1)	2 (3.2)

**Table 2:** Best Overall Response Rate per RECIST v1.1 in Advanced Myxoid/Round Cell Liposarcoma. ^a^ Per RECIST v1.1; ^b^ CI calculated with the two-sided Clopper–Pearson exact binomial method [31]. Abbreviations: CI, confidence interval; n, number with response; N, total population evaluable for response assessment; RECIST, Response Evaluation Criteria in Solid Tumors.

	n/N (%)	95% CI ^b^
Objective response rate ^a^	8/49 (16.3)	7.3–29.7
Disease control rate	38/49 (77.6)	63.4–88.2
Best overall response		
Partial response	8/49 (16.3)	-
Stable disease	30/49 (61.2)	-
Progressive disease	11/49 (22.4)	-

## Data Availability

The original proposal approved by the Yael University Open Data Access Project can be found at: https://yoda.yale.edu/data-request/2024-0560/, accessed on 17 June 2024. The data is held by the Yale University Open Data Access Project, and all data results are subject to review and approval by the YODA project.

## Abbreviations

The following abbreviations have been used in this manuscript:

CDK4	Cyclin-Dependent Kinase 4
CHOP	C/EBP Homologous Protein
CI	Confidence Interval
*DDIT3*	DNA Damage-Inducible Transcript 3 gene
DNA	deoxyribose nucleic acid
*EWSR1*	Ewing Sarcoma Breakpoint Region 1 gene
*FUS*	Fused in Sarcoma gene
HR	Hazard Ratio
LPS	Liposarcoma
MDM2	Mouse Double Minute 2
MRCLS	Myxoid/Round Cell Liposarcoma
ORR	Objective Response Rate
OS	Overall Survival
Q3W	Every 3 weeks
RECIST	Response Evaluation Criteria in Solid Tumors
STS	Soft Tissue Sarcoma
YODA	Yale University Open Data Access
